# Lipid droplets in *Arabidopsis thaliana* leaves contain myosin-binding proteins and enzymes associated with furan-containing fatty acid biosynthesis

**DOI:** 10.3389/fpls.2024.1331479

**Published:** 2024-03-01

**Authors:** Yuto Omata, Reina Sato, Emi Mishiro-Sato, Keiko Kano, Haruko Ueda, Ikuko Hara-Nishimura, Takashi L. Shimada

**Affiliations:** ^1^ Faculty of Horticulture, Chiba University, Matsudo, Japan; ^2^ World Premier International Research Center Initiative-Institute of Transformative Bio-Molecules (WPI-ITbM), Nagoya University, Nagoya, Japan; ^3^ Faculty of Science and Engineering, Konan University, Kobe, Japan; ^4^ Graduate School of Horticulture, Chiba University, Matsudo, Japan; ^5^ Plant Molecular Science Center, Chiba University, Chiba, Japan; ^6^ Research Center for Space Agriculture and Horticulture, Chiba University, Matsudo, Japan

**Keywords:** lipid droplet proteomics, myosin-binding protein, enzymes for furan-containing fatty acid biosynthesis, actin filament, endoplasmic reticulum, *Arabidopsis thaliana*

## Abstract

Lipid droplets (LDs) are lipid storage organelles in plant leaves and seeds. Seed LD proteins are well known, and their functions in lipid metabolism have been characterized; however, many leaf LD proteins remain to be identified. We therefore isolated LDs from leaves of the leaf LD–overaccumulating mutant *high sterol ester 1* (*hise1*) of *Arabidopsis thaliana* by centrifugation or co-immunoprecipitation. We then performed LD proteomics by mass spectrometry and identified 3,206 candidate leaf LD proteins. In this study, we selected 31 candidate proteins for transient expression assays using a construct encoding the candidate protein fused with green fluorescent protein (GFP). Fluorescence microscopy showed that MYOSIN BINDING PROTEIN14 (MYOB14) and two uncharacterized proteins localized to LDs labeled with the LD marker. Subcellular localization analysis of MYOB family members revealed that MYOB1, MYOB2, MYOB3, and MYOB5 localized to LDs. LDs moved along actin filaments together with the endoplasmic reticulum. Co-immunoprecipitation of myosin XIK with MYOB2-GFP or MYOB14-GFP suggested that LD-localized MYOBs are involved in association with the myosin XIK–LDs. The two uncharacterized proteins were highly similar to enzymes for furan fatty acid biosynthesis in the photosynthetic bacterium *Cereibacter sphaeroides*, suggesting a relationship between LDs and furan fatty acid biosynthesis. Our findings thus reveal potential molecular functions of LDs and provide a valuable resource for further studies of the leaf LD proteome.

## Introduction

Lipids are important as energy sources, membrane components, and signal transducers in plants. Excess lipids are converted to neutral lipids, including triacylglycerols and sterol esters, and are stored in lipid droplets (LDs) (Grimberg et al., 2015; Shimada et al., 2019). LDs are formed on the endoplasmic reticulum (ER). In plants, triacylglycerols are produced by acyl-CoA:diacylglycerol acyltransferase (DGAT) or phosphatidylcholine:diacylglycerol acyltransferase (PDAT), which are localized on the ER (Shockey et al., 2006; Kim et al., 2011). Sterol esters are produced by phospholipid:sterol acyltransferase (PSAT) or acyl-CoA:sterol acyltransferase (ASAT), which are also localized on the ER (Lara et al., 2018; Shimada et al., 2019). In *Arabidopsis thaliana*, deficiency of DGAT, PDAT, or PSAT inhibits LD formation (Shimada et al., 2019; Bose et al., 2021). LDs are abundant in seed cells of oilseed plants but are also present in leaf cells. Leaf LD formation can be induced by heat stress, cold stress, and phosphate starvation (Shimojima et al., 2015; Gidda et al., 2016). In addition, certain mutants and transformants overaccumulate LDs in leaves (James et al., 2010; Shimada et al., 2019; Vanhercke et al., 2019). For instance, HISE1 down-regulates the content of 3-hydroxy-3-methylglutaryl-coenzyme A reductase (HMGR), a rate-limiting enzyme in sterol biosynthesis (Shimada et al., 2019), and leaves of the *A. thaliana* mutant *high sterol ester 1* (*hise1*) overaccumulate LDs that mainly contain sterol esters (Shimada et al., 2019). Such LD-overaccumulating lines can facilitate the characterization and isolation of LDs.

It was previously assumed that LDs serve only for carbon storage; however, recent studies have revealed LD-localized proteins with unique functions. Members of three protein families, oleosins, caleosins, and steroleosins, have been identified as seed LD proteins (Chapman et al., 2012). Oleosins have an important role in inhibiting LD fusion (Siloto et al., 2006), contributing to freezing tolerance of seeds and facilitating normal germination (Shimada et al., 2008). Leaf LD proteins, including caleosin 3 and α-dioxygenase 1 (α-DOX1), have recently been identified by LD proteomics in *A. thaliana* leaves (Yang et al., 2012; Shimada et al., 2014; Brocard et al., 2017; Fernandez-Santos et al., 2020; Kretzschmar et al., 2020). Caleosin 3 and α-DOX1 are involved in the production of 2-hydroxy-octadecatrienoic acid, which has antifungal activity against members of the genus *Colletotrichum* (Shimada et al., 2014), suggesting that leaf LDs contribute to the defense response against fungi. LD-associated proteins (LDAPs), including LDAP3, are highly similar to small rubber particle proteins identified in *Hevea brasiliensis* and are found in leaf LDs of plants (Gidda et al., 2013; Horn et al., 2013). LDAPs mediate LD size and contribute to drought tolerance (Gidda et al., 2016; Kim et al., 2016). LDAPs of *A. thaliana* interact with LDAP-INTERACTING PROTEIN (LDIP), which is also a leaf LD protein (Pyc et al., 2017). In addition, some lipases, GLYCEROL-3-PHOSPHATE ACYLTRANSFERASE4 (GPAT4), GPAT8, and CYCLOARTENOL SYNTHASE1 are localized in leaf LDs (Fernandez-Santos et al., 2020). These findings suggest that leaf LDs contain various proteins with important functions; however, few leaf LD proteins have been identified and characterized. To resolve the molecular and physiological functions of leaf LDs, identification of additional leaf LD proteins is necessary.

Previous leaf LD proteomics efforts have used leaf LDs induced by senescence or pathogen infection (Shimada et al., 2014); however, the LD yield from these approaches is low. Here, we used *A. thaliana hise1* mutant leaves, which stably accumulate high levels of LDs that are easy to isolate. We performed leaf LD proteomics by mass spectrometry analysis and identified many candidate leaf LD proteins. These included five myosin-binding proteins (MYOBs) and two putative enzymes for furan-containing fatty acid biosynthesis. MYOBs are closely related to myosin and its function, and their localization in LDs represents a breakthrough in elucidating LD movement by myosin. In addition, identification of putative enzymes for furan-containing fatty acid biosynthesis provides insight into plant lipid metabolism by leaf LDs.

## Materials and methods

### Plant materials and growth conditions

*A. thaliana* accession Columbia (Col-0) was used as the wild type. The *A. thaliana* T-DNA mutant *hise1-2* (Shimada et al., 2019), plants containing Lifeact–Venus driven by the cauliflower mosaic virus 35S promoter (Era et al., 2009), and plants containing myosin XIK fused with yellow fluorescent protein (YFP) driven by the *myosin XIK* promoter (XIK-YFP plants) (Peremyslov et al., 2012) were in the Col-0 background. Transgenic *A. thaliana* expressing cytosolic GFP was used as a negative control in the Co-IP experiment (Mano et al., 2002). Seeds were surface sterilized with 70% (v/v) ethanol, dried, and sown on Murashige and Skoog (MS) agar plates (Wako, Tokyo, Japan) containing 1% (w/v) sucrose. The plates were incubated at 23°C under continuous light (100 µE s^–1^ m^–2^) for 4 weeks. Seedlings were transplanted to vermiculite and grown at 23°C under continuous light (100 µE s^–1^ m^–2^) for 8 weeks.

### Vector construction

Vectors were constructed using Gateway Technology (Invitrogen, Carlsbad, CA) with the destination vectors pGWB404m (Nakagawa et al., 2007; Segami et al., 2014), pGWB405m (Nakagawa et al., 2007; Segami et al., 2014), and pGWB406m (Nakagawa et al., 2007; Segami et al., 2014).

The *LDAP3* genomic DNA fragment (nucleotides –1,799 to +1,263) was PCR amplified from *A. thaliana* cDNA (template) using the primer set 5′-CACCAGAAGATGAGTCACTTGAATT-3′ and 5′-ATCAAGTGGATGGAACTCCAA-3′. The PCR product was cloned into pENTR/D-TOPO (Invitrogen) via TOPO cloning to produce the subcloning vector pENTR-*proLDAP3*:*LDAP3*. The vector pENTR-*proLDAP3*:*LDAP3* was transferred into the destination vector pGWB404m through an LR recombination reaction (Invitrogen) to create the vector pGWB404m-*proLDAP3:LDAP3*.

At1g19310 (+1 to +687 bp, where +1 bp refers to the transcription start site) without the stop codon was amplified by PCR from *A. thaliana* cDNA using the primer set 5′-CACCATGTCTGATGTCCCTTCTTG-3′ and 5′-GCTCAAGAAGAGAGAAAACA-3′. The PCR product was cloned into pENTR/D-TOPO (Invitrogen) via TOPO cloning to produce the subcloning vector pENTR-19310. The pENTR-19310 vector was digested with the restriction enzymes *Asc*I and *Not*I. The digested DNA fragment (2,555 bp, pENTR-CUT) was purified and used for subcloning.

*MYOB* genes, AT3G23510, AT1G30130, AT4G33110, the globular tail domain of myosin XIK (XIKGTD), and other constructs (described in **Supplementary Figure S1**) were PCR amplified using primer sets (FWD and REV) provided in **Supplementary Table S4**. To produce entry clones, the PCR products were ligated into pENTR-CUT using an In-Fusion Cloning Kit (Takara, Shiga, Japan). The entry clones were cloned into the destination vector pGWB405m (for C-terminal fusion with mGFP) or pGWB406m (for N-terminal fusion with mGFP) using LR Clonase II (Invitrogen) to create expression vectors for cellular localization.

### Transient expression assay using *Nicotiana benthamiana* leaves

Transient expression assays were performed as described previously (Shimada et al., 2019). The expression vectors were independently transformed into Agrobacterium (*Agrobacterium tumefaciens*) strain GV3101, and the transformed Agrobacterium was infiltrated into the leaves of 3-week-old *N*. *benthamiana* plants. The expression vector containing *α-DOX1-GFP* or *α-DOX1-RFP* was used as a LD marker (Shimada et al., 2019). One day post-infiltration, leaves were soaked in 3 mM mevalonate solution for 1 day to induce LDs. Two days post-infiltration, the leaves were observed under a fluorescence microscope (BZ-X800; Keyence, Osaka, Japan). GFP fluorescence was examined with a GFP filter (excitation: 450–490 nm; emission: 500–550 nm), and RFP fluorescence was examined with a TRITC filter (excitation: 520–570 nm; emission: 570–640 nm).

### Observation of organelle movements

Two-week-old Lifeact–Venus plants (wild-type background) were soaked in 3 mM mevalonate solution for 1 day to induce LDs. LDs in the epidermal cells of true leaves were stained with 5 µg/mL BODIPY 493/503 (Thermo Fisher Scientific, Waltham, MA) (Fernandez-Santos et al., 2020) and observed under a fluorescence microscope (BZ-X800). The fluorescent signals of Lifeact–Venus and BODIPY 493/503 were examined with a GFP filter (excitation: 450–490 nm; emission: 500–550 nm). Punctate structures emitting fluorescence were defined as LDs, and filament structures were defined as actin filaments.

Expression vectors containing *α-DOX1-RFP* (Shimada et al., 2014) and *mCherry-HDEL* (Nelson et al., 2007) were used as LD and ER markers, respectively. Agrobacterium containing the respective constructs was infiltrated into the leaves of 3-week-old *N*. *benthamiana* plants. One day post-infiltration, leaves were soaked in 3 mM mevalonate solution for 1 day to induce LDs. Two days post-infiltration, the epidermal cells of true leaves were observed under a fluorescence microscope (BZ-X800). Fluorescent signals of RFP and mCherry were examined with a TRITC filter (excitation: 520–570 nm; emission: 570–640 nm). Punctate structures emitting fluorescence were defined as LDs, while network structures were defined as ER.

### Stable transformation of *A. thaliana* plants

The vector pGWB404m-*proLDAP3*:*LDAP3* was introduced into *hise1-2 A. thaliana* plants via Agrobacterium-mediated transformation (strain GV3101) using the floral dip method (Clough and Bent, 1998) to express the LDAP3-GFP fusion protein under the control of the *LDAP3* promoter (*pLDAP3*:*LDAP3-GFP* plants).

Expression vectors containing *MYOB2* or *MYOB14* in pGWB405m (named pGWB405m-*MYOB2* and pGWB405m-*MYOB14*, respectively) were introduced into wild-type *A. thaliana* plants via Agrobacterium-mediated transformation (strain GV3101) using the floral dip method (Clough and Bent, 1998) to express the MYOB2-GFP and MYOB14-GFP fusion proteins, respectively. True leaves of 2-week-old plants were treated with 3 mM mevalonate treatment for 1 day, and then the epidermal cells were observed under a fluorescence microscope (BZ-X800).

### Centrifuge method for LD isolation

To isolate LDs from *A. thaliana* leaves, 3.5 g of leaves from 4-week-old plants (wild type or *hise1-2*) was homogenized in 5 mL G-20 buffer (100 mM Tris-HCl pH 7.5, 10 mM KCl, 1 mM EDTA, and 20% [w/v] sucrose) using a pestle and mortar. The homogenates were filtered through two layers of cheese cloth and centrifuged for 10 min at 10,000 *g* at 4°C. Supernatants (1.5 mL) were collected in fresh 2-mL tubes. The resuspensions were covered with 0.2 mL of G-15 buffer (100 mM Tris-HCl pH 7.5, 10 mM KCl, 1 mM EDTA, and 15% [w/v] sucrose) and 0.4 mL of G-5 buffer (100 mM Tris-HCl pH 7.5, 10 mM KCl, 1 mM EDTA, and 5% [w/v] sucrose) and centrifuged for 10 min at 20,000 *g* at 4°C. Floating lipid pads (0.3 mL) were collected in fresh 2-mL tubes. The resuspensions were covered with 0.4 mL of G-0 buffer (100 mM Tris-HCl pH 7.5, 10 mM KCl, and 1 mM EDTA) and centrifuged for 10 min at 20,000 *g* at 4°C. Floating lipid pads (0.1 mL) were collected in fresh 2-mL tubes and defined as isolated LDs. The isolated LDs were stained with 5 µg/mL BODIPY 493/503 (Thermo Fisher Scientific). Fluorescence was examined under a fluorescence microscope (BZ-X800) with a GFP filter (excitation: 450–490 nm; emission: 500–550 nm).

### Co-immunoprecipitation method for LD isolation

The Co-IP LD isolation method was performed using a μMACS GFP-tagged protein isolation kit (Miltenyi Biotec, Gladbach, Germany) following the method of a previous study (Shimada et al., 2014). Four-week-old *hise1-2*/*pLDAP3:LDAP3-GFP* plants (0.5 g fresh weight) and wild-type plants expressing cytosolic GFP alone were ground with 1.5 mL extraction buffer containing 20 mM Tris-HCl (pH 7.5), 150 mM NaCl, 1 mM CaCl_2_, and 1 mM MgCl_2_ using a pestle and mortar. The extracts were centrifuged at 1,000 *g* for 5 min at 4°C followed by 8,000 *g* for 10 min at 4°C. The supernatants (1 mL each) were subjected to immunoprecipitation with 50 μL of anti-GFP microbeads (μMACS GFP-tagged protein isolation kit). The samples were incubated at 4°C for 30 min and applied to a μ column (Miltenyi Biotec). The column was washed with 1 mL of the extraction buffer. Pure immunoprecipitates were eluted with 50 μL of sample buffer (100 mM Tris-HCl pH 6.8, 4% [w/v] SDS, 12% [v/v] 2-mercaptoethanol, and 20% [v/v] glycerol) and defined as isolated LDs.

### Mass spectrometry analysis of isolated LDs

Immunoprecipitated samples were dissolved in sample buffer and resolved (~2 cm) using SDS-PAGE. Each lane was divided into six pieces. In-gel digestion was performed according to a method described previously (Kano et al., 2023). The digested peptides were analyzed by nano-flow reverse-phase LC followed by tandem MS using a Q-Exactive hybrid mass spectrometer (Thermo Fisher Scientific). The capillary reverse-phase HPLC–MS/MS system comprised a Dionex U3000 gradient pump equipped with a VICI CHEMINERT valve. The Q-Exactive mass spectrometer was equipped with a nano-electrospray ionization (NSI) source (AMR, Tokyo, Japan). The desalted peptides were loaded into a separation capillary C18 reverse-phase column (NTCC-360/100–3–125, 125 × 0.1 mm, Nikkyo Technos). Peptide spectra over a mass range of m/z 350–1,800 were recorded using an Xcalibur 3.0.63 system (Thermo Fisher Scientific). MS spectra were subsequently recorded followed by 10 data-dependent high-energy collisional dissociation (HCD) MS/MS spectra generated from the 10 highest intensity precursor ions. MS/MS spectra were interpreted and peak lists were generated using Proteome Discoverer 2.2.0.388 (Thermo Fisher Scientific). Searches were performed using SEQUEST (Thermo Fisher Scientific) against the TAIR10 and cRAP for contaminant databases. Search parameters were set as follows: enzymes selected with a maximum of two missing cleavage sites, a mass tolerance of 10 ppm for peptide tolerance, 0.02 Da for MS/MS tolerance, fixed modification of carbamidomethyl (C), and variable modification of oxidation (M). Peptide identifications were based on significant Xcorr values (high confidence filter). Peptide identification and modification information returned from SEQUEST were manually inspected and filtered to obtain confirmed peptide identification and modification lists from HCD MS/MS.

### LD staining with MDH in plants

*A. thaliana* leaves of 4-week-old *hise1-2*/*pLDAP3*:*LDAP3-GFP* plants were immersed into 2 µM monodansylpentane (MDH; AUTODOT Visualization Dye, Abcepta, San Diego, CA) for 1 min. Fluorescence was examined under a fluorescence microscope (BZ-X800) with a DAPI filter (excitation: 340–380 nm; emission: 435–485 nm). The fluorescent signal from LDAP3-GFP was examined using a GFP filter (excitation: 450–490 nm; emission: 500–550 nm).

The hypocotyls of 4-day-old *A. thaliana* XIK-YFP plants were soaked in 3 mM mevalonate solution for 1 day to induce LD formation. Fluorescence was examined under a fluorescence microscope (BZ-X800) with a DAPI filter (excitation: 340–380 nm; emission: 435–485 nm). The fluorescent signal from XIK-YFP was examined using a GFP filter (excitation: 450–490 nm; emission: 500–550 nm).

### Co-immunoprecipitation using MYOB2-GFP and MYOB14-GFP plants

Co-IP assays were performed using a μMACS GFP-tagged protein isolation kit (Miltenyi Biotec). Three-week-old *A. thaliana* plants (0.5 g fresh weight) expressing *MYOB2-GFP* or *MYOB14-GFP* were ground in 1 mL of lysis buffer (150 mM NaCl, 1% [v/v] Ecosurf EH-9, and 50 mM Tris-HCl pH 8.0). The extracts were centrifuged at 1,000 × *g* for 1 min at 4°C. This was followed by centrifugation of the supernatants at 15,000 × *g* for 10 min at 4°C. A 1-mL aliquot of each supernatant was subjected to Co-IP with 50 μL of anti-GFP microbeads. Samples were incubated at 4°C for 30 min and applied to a μ column (Miltenyi Biotec). The column was washed with 1 mL of lysis buffer and 0.2 mL of 20 mM Tris-HCl, pH 7.5. Pure immunoprecipitates were eluted with 50 μL of sample buffer (100 mM Tris-HCl pH 6.8, 4% [w/v] SDS, 12% [v/v] 2-mercaptoethanol, and 20% [v/v] glycerol).

### Mass spectrometry analysis of pure immunoprecipitate samples from MYOB2-GFP and MYOB14-GFP plants

Mass spectrometry analysis was outsourced to BGI (Shenzhen, China). To identify proteins in the pure immunoprecipitates from MYOB2-GFP and MYOB14-GFP plants, the samples were separated by gel electrophoresis. Proteins in the samples were digested using trypsin and separated by Thermo UltiMate 3000 UHPLC. The separated peptides were ionized using a nanoESI source and analyzed by a Q-Exactive HF X tandem mass spectrometer (Thermo Fisher Scientific) in data-dependent acquisition mode. The ion source voltage was set to 1.9 kV, MS1 scanning range was 350–1,500 m/z, resolution was set to 60,000, MS2 starting m/z was fixed at 100, and resolution was 15,000. The UniProt protein database (https://www.uniprot.org/) was used for protein profiling.

### Silver staining

Immunoprecipitates were subjected to SDS-PAGE on a 5–20% acrylamide-gradient gel (SuperSep Ace, Fuji film, Tokyo, Japan). Silver staining was performed using a Silver Stain MS Kit (Wako, Tokyo, Japan).

### Immunoblotting

Immunoprecipitates were subjected to SDS-PAGE on a 5–20% acrylamide-gradient gel (SuperSep Ace). The proteins were electrophoretically transferred from the gel onto a polyvinylidene difluoride membrane (PVDF; GE Healthcare, Chicago, IL). The membrane was treated with blocking solution (5% [w/v] skim milk, Tris-HCl pH 7.5, and 0.1% [v/v] Triton X) for 30 min and treated with anti-GFP antibody (1:10,000; JL-8, Takara, Shiga, Japan) at 4°C for 24 h. Horseradish peroxidase–conjugated anti-mouse IgG (1:2,000) was used as the secondary antibody. Immunodetection was performed with a chemiluminescent system using ImmunoStar LD (Wako) and a C-DiGit blot scanner (LI-COR, Lincoln, NE).

### Accession numbers

The plant genes mentioned in this study can be found in the GenBank/EMBL databases under the following accession numbers: *LDAP3* (AT3G05500), *AtUFAMD1* (AT3G23510), *AtUFAO1* (AT1G30130), *AtFUFM1/LIME1* (AT4G33110), *MYOB1* (AT1G08800), *MYOB2* (AT1G70750), *MYOB3* (AT5G16720), *MYOB4* (AT2G30690), *MYOB5* (AT1G18990), *MYOB6* (AT1G74830), *MYOB7* (AT5G06560), *MYOB8* (AT3G11850), *MYOB9* (AT3G54740), *MYOB10* (AT3G30830), *MYOB12* (AT5G57830), *MYOB13* (AT4G13630), *MYOB14* (AT4G13160), *MYOB15* (AT1G04890), *MYOB16* (AT1G18265), and *myosin XIK* (AT5G20490). The proteins of *C. sphaeroides* mentioned in this study can be found in UniProt (The UniProt, 2017) under the following accession numbers: UfaO (Q3IYV8), FufM (Q6NBA4), UfaM (Q3J4I7), and UfaD (Q3IYV7).

## Results

### Proteomic analysis of LDs isolated from *hise1* mutant leaves

**Figure 1:**
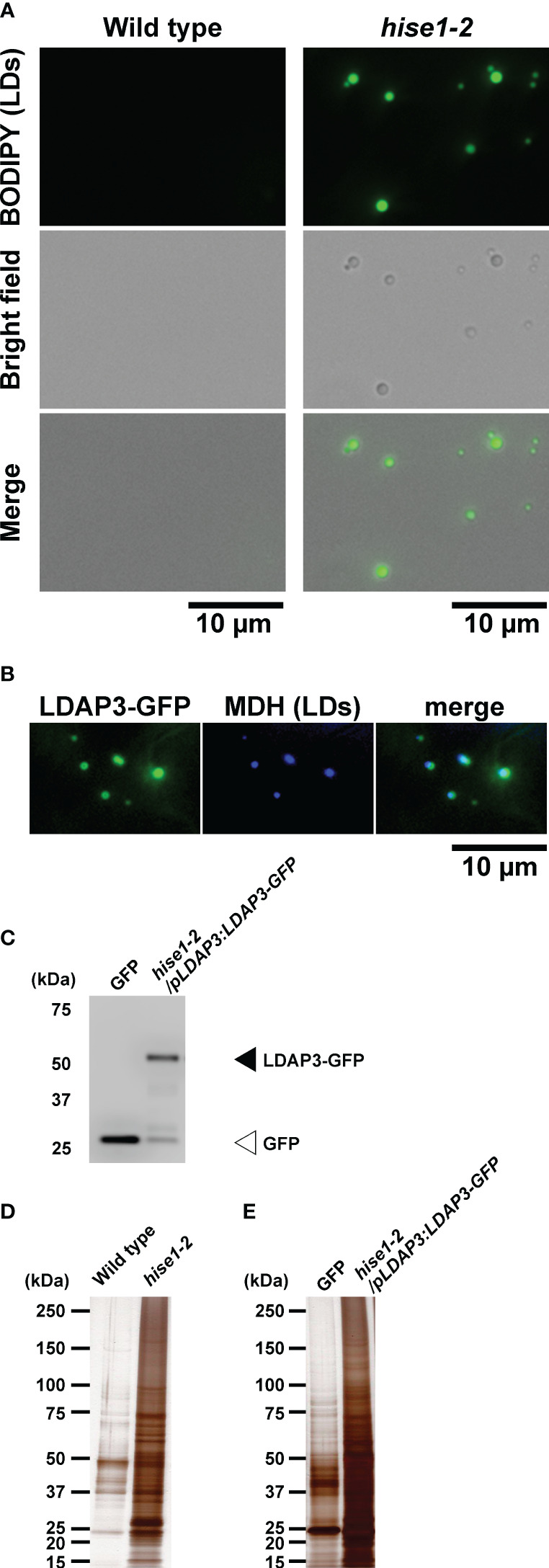
Isolation of leaf LDs from *A thaliana* leaves using centrifuge and co-immunoprecipitation (Co-IP) methods. **(A)** BODIPY fluorescence and brightfield images of LDs isolated from the wild type and *hise1-2* mutant using a centrifuge method and stained with BODIPY 493/503. **(B)** Fluorescence images of LDAP3-GFP and MDH (LDs) in epidermal cells of *hise1-2*/*pLDAP3*:*LDAP3-GFP* plants. **(C)** Immunoblot analysis of the co-immunoprecipitants of *hise1-2*/*pLDAP3:LDAP3-GFP* plants, and wild-type plants expressing cytosolic GFP alone, with anti-GFP antibody. **(D, E)** Silver staining of the co-immunoprecipitants from the centrifuge method **(D)** and the Co-IP method **(E)**.

To perform leaf LD proteomics, we isolated LDs from leaves of the *A. thaliana hise1-2* mutant via a centrifuge method and a co-immunoprecipitation (Co-IP) method. LDs rise to the top during centrifugation (Shimada et al., 2008), so we centrifuged leaf extracts from wild-type or *hise1-2* plants and collected the top fraction. Microscopy observation showed that the top fraction from the *hise1-2* mutant contained LDs (**Figure 1A**). By contrast, we could not collect the top fraction from wild-type leaves (used as a negative control), which have few LDs. We labeled the fraction from the *hise1* mutant as isolated leaf LDs. For Co-IP analysis with anti-GFP antibodies, we used a transgenic *A. thaliana hise1-2* mutant expressing a LDAP3-GFP fusion under control of the *LDAP3* promoter (*hise1-2*/*pLDAP3:LDAP3-GFP* plants). Fluorescence microscopy revealed that LDAP3-GFP was localized to LDs in epidermal cells of *hise1-2* leaves (**Figure 1B**). Transformants expressing cytosolic GFP (Mano et al., 2002) were used as a negative control because cytosolic GFP does not fuse with LDs. Because we detected LDAP3-GFP in the co-immunoprecipitants from *hise1-2*/*pLDAP3:LDAP3-GFP* plants (**Figure 1C**), we labeled the co-immunoprecipitants as isolated leaf LDs. Silver staining revealed that the isolated leaf LDs contained a lot of protein (**Figures 1D, E**). We detected 3,206 proteins in the isolated LDs, but not in the negative controls, using mass spectrometry (**Supplementary Table S1**). We defined these as candidate leaf LD proteins (**Supplementary Table S1**, LD candidates = Yes).

### Subcellular localization of 31 candidate leaf LD proteins

**Figure 2:**
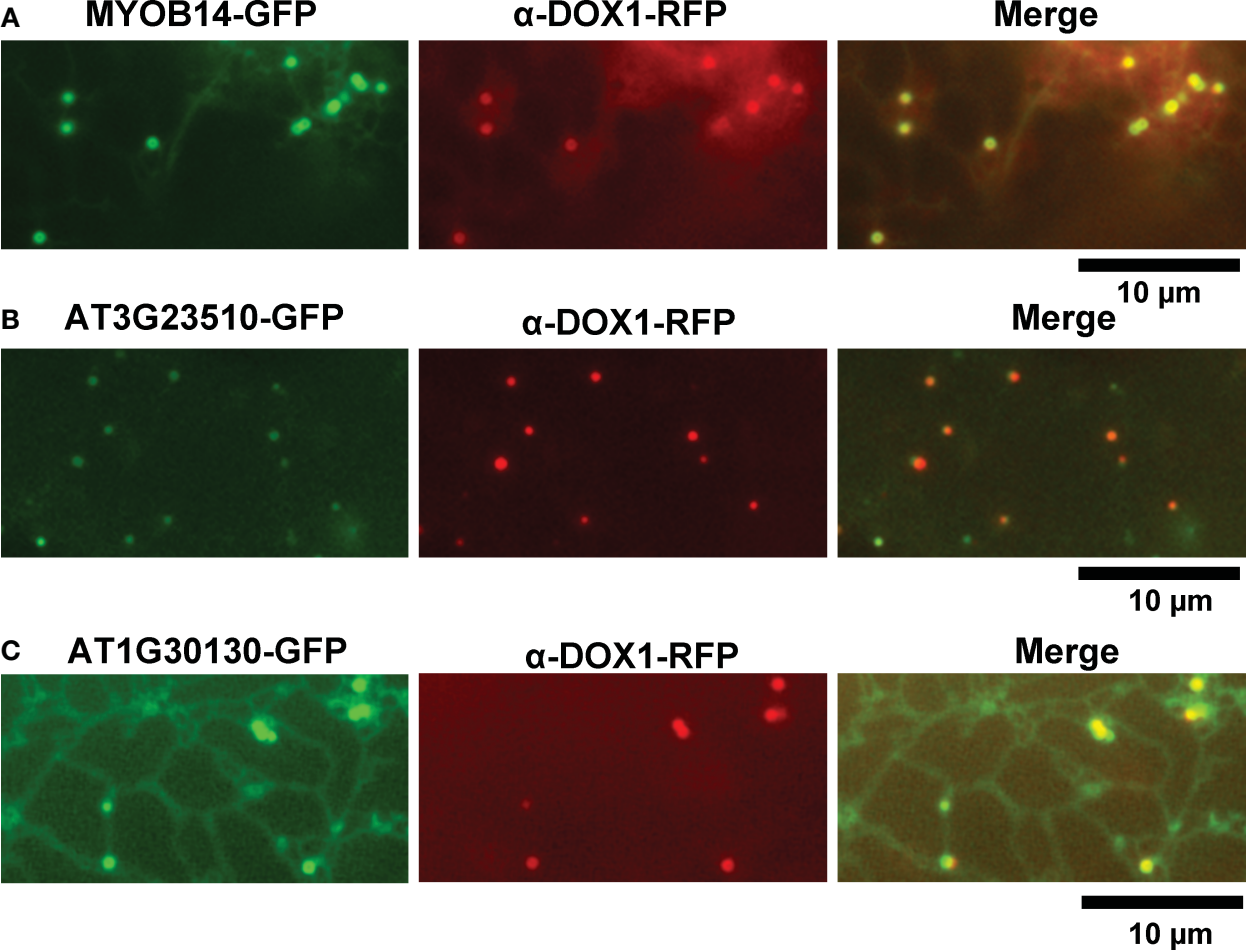
Subcellular localization of MYOB14, AT3G23510, and AT1G30130. **(A–C)** Fluorescence images of *N. benthamiana* leaves transiently expressing MYOB14-GFP **(A)**, AT3G23510-GFP **(B)**, or AT1G30130-GFP **(C)** along with α-DOX1-RFP (LD marker).

We selected 31 proteins related to lipid metabolism or ER function from the leaf LD protein candidates. To investigate their subcellular localization, we transiently expressed each candidate protein fused with GFP or red fluorescent protein (RFP) in leaves of *Nicotiana benthamiana*. We induced leaves to form LDs with mevalonate treatment and visualized them using α-DOX1-RFP or BODIPY 493/503 staining. MYOB14-GFP fluorescence was associated with punctate structures (**Figure 2A**). The punctate structures co-localized with α-DOX1-RFP (**Figure 2A**), indicating that MYOB14-GFP was localized to LDs. Two uncharacterized proteins (encoded by AT3G23510 and AT1G30130) also co-localized with α-DOX1-RFP, indicating LD localization (**Figures 2B, C**). A domain search using InterProScan (Paysan-Lafosse et al., 2023) revealed that AT3G23510 encodes a protein classified as cyclopropane-fatty-acyl-phospholipid synthase, and AT1G30130 encodes a protein that contains domain of unknown function 1365 (DUF1365). To our knowledge, this is the first report describing LD localization of these three proteins. The other 28 proteins were not localized to LDs (**Supplementary Figure S1**).

### Subcellular localization of 14 MYOBs, including 5 LD-localized MYOBs

**Figure 3:**
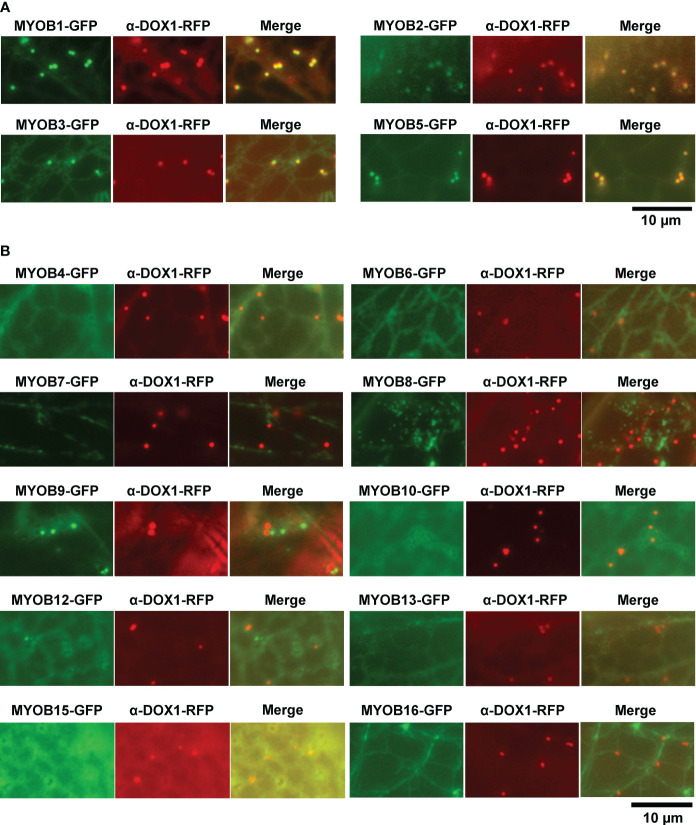
Subcellular localization of MYOB1, MYOB2, MYOB3, and MYOB5. **(A)** Fluorescence images of *N. benthamiana* leaves transiently expressing MYOB1-GFP, MYOB2-GFP, MYOB3-GFP, or MYOB5-GFP along with α-DOX1-RFP. **(B)** Fluorescence images of *N. benthamiana* leaves transiently co-expressing α-DOX1-RFP and the other MYOB proteins tested in this study.

MYOBs interact with myosin through the DUF593 domain (Peremyslov et al., 2013). *A. thaliana* contains 16 MYOBs (Peremyslov et al., 2013; Perico et al., 2021), but their LD localization is unknown. To investigate the LD localization of *A. thaliana* MYOBs, we fused each of the 14 MYOBs with GFP and transiently expressed them in *N. benthamiana* leaves. The fluorescence of MYOB1-GFP, MYOB2-GFP, MYOB3-GFP, and MYOB5-GFP co-localized with that of α-DOX1-RFP (**Figure 3A**), while the other MYOBs did not appear to localize to LDs (**Figure 3B**). Our results indicate that MYOB1, MYOB2, MYOB3, and MYOB5 also localize to LDs.

### Leaf LDs move along actin filaments together with the ER

**Figure 4:**
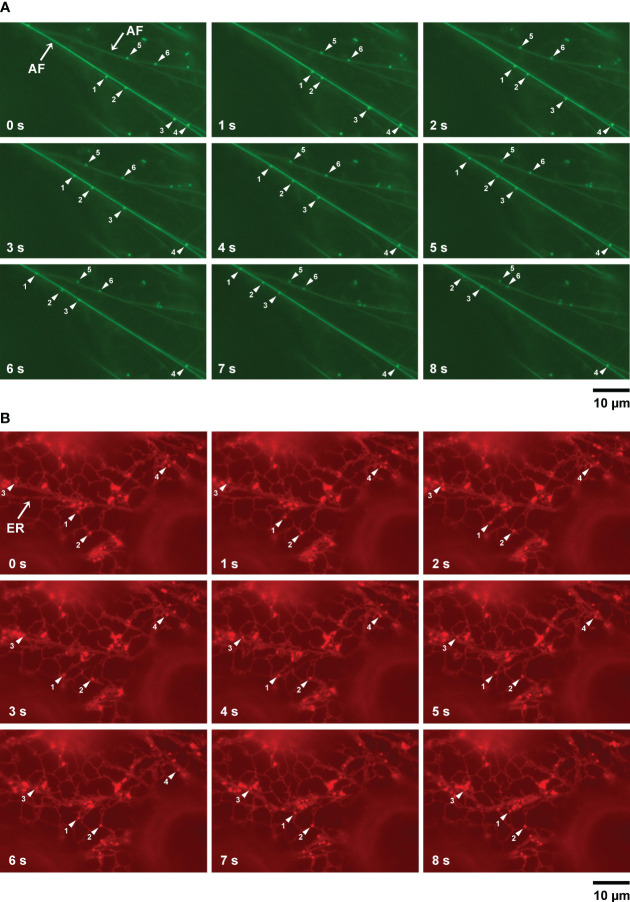
LD movement on actin filaments and the ER captured by time-lapse imaging. **(A)** Fluorescence images of epidermal cells of true leaves of 2-week-old *A thaliana* plants expressing Lifeact–Venus and stained with BODIPY 493/503 after inducing LDs with mevalonic acid. Lifeact–Venus, actin filament (filamented structures); BODIPY 493/503, LDs (punctate structures). Arrowheads show LDs (same numbers indicate same LDs). **(B)** Fluorescence images of epidermal cells of true leaves of *N. benthamiana* transiently co-expressing α-DOX1-RFP (LDs, punctate structures) and mCherry-HDEL (ER, network structures). Arrowheads show LDs (same numbers indicate same LDs).

Myosin functions in the movement of organelles in association with actin filaments. MYOBs work together with myosin to regulate organelle movement (Peremyslov et al., 2015; Perico et al., 2021). To determine if there is a relationship between LD movement and actin filaments, we used *A. thaliana* plants (wild-type background) producing Lifeact–Venus, which localizes to actin filaments (Era et al., 2009). After inducing LDs through treatment with mevalonic acid, we co-visualized LDs and actin filaments in the epidermal cells of true leaves stained with BODIPY 493/503. LDs moved along actin filaments and sometimes paused (**Figure 4A**; **Supplementary Movie S1**).

ER morphology is involved in actin filament organization (Ueda et al., 2010). To observe the relationship between LD movement and the ER, we co-visualized LDs and the ER in epidermal cells of true leaves of *N. benthamiana* carrying the LD marker α-DOX1-RFP and the ER marker mCherry-HDEL, after induction of LDs using mevalonic acid. LDs moved together with the ER and sometimes paused, similar to their movement on actin filaments (**Figure 4B**; **Supplementary Movie S2**). In addition, we co-visualized LDs and the ER in epidermal cells of true leaves of *N. benthamiana* carrying the LD marker α-DOX1-GFP and the ER marker mCherry-HDEL, after induction of LDs with mevalonic acid. Almost all LDs co-localized with the ER (**Supplementary Figure S2**). These results suggest that LDs move along actin filaments together with the ER.

### LD-localized MYOBs interact with myosin XIK

Myosin XIK is a major myosin XI in *A. thaliana* and interacts with MYOB1, MYOB2, and MYOB3 (Peremyslov et al., 2013; Kurth et al., 2017); the DUF593 domain of MYOBs binds to the globular tail domain of myosin. However, the proteins interacting with MYOB14 are unclear, and there are no interactome data using LD-localized MYOBs. We therefore performed Co-IP using MYOB2 or MYOB14 fused with GFP (MYOB2-GFP and MYOB14-GFP). We generated transgenic *A. thaliana* plants expressing *MYOB2-GFP* or *MYOB14-GFP* (**Supplementary Figures S3A, B**). Co-immunoprecipitants were prepared from leaves of the transgenic *A. thaliana* plants using anti-GFP antibodies (**Supplementary Figures S3C, D**). We performed mass spectrometry analysis of the co-immunoprecipitants and identified candidate proteins that interact with MYOB2 and MYOB14, including myosin XIK (**Supplementary Tables S2**, **S3**). In addition, we identified some candidates of interactors with MYOB2 of MYOB14, suggesting that MYOB2 and MYOB14 interact with myosin XIK and other proteins.

To investigate whether myosin XIK localizes to LDs, we co-expressed the GTD domain of myosin XIK (XIKGTD) fused with GFP and the LD marker α-DOX1-RFP in *N. benthamiana*. We then observed fluorescence of GFP-XIKGTD and α-DOX1-RFP in epidermal cells of true leaves after induction of LDs with mevalonic acid. GFP-XIKGTD fluorescence was observed in the cytosol and did not co-localize with LDs (**Supplementary Figure S4A**). In addition, we observed hypocotyls of *A. thaliana* XIK-YFP plants (Peremyslov et al., 2012) following the induction of LD formation with mevalonic acid. This YFP-tagged myosin XIK is previously shown to be a functional myosin (Peremyslov et al., 2012; Okamoto et al., 2015). Fluorescence microscopy showed XIK-YFP signals in a filamentous pattern (**Supplementary Figure S4B**), as previously observed (Peremyslov et al., 2012). The LDs co-localized with the filamentous XIK-YFP signals. Notably, however, the XIK-YFP fluorescence signals did not resemble LD-like punctate structures (**Supplementary Figure S4B**), although MYOB1, MYOB2, MYOB3, and MYOB5 localized to LDs (**Figures 2A**, **3A**). These results suggest that binding between myosin XIK and LD-localized MYOBs may occur only at the contact sites between LDs and actin filaments.

### Enzymes involved in biosynthesis of furan-containing fatty acids localize to LDs

**Figure 5:**
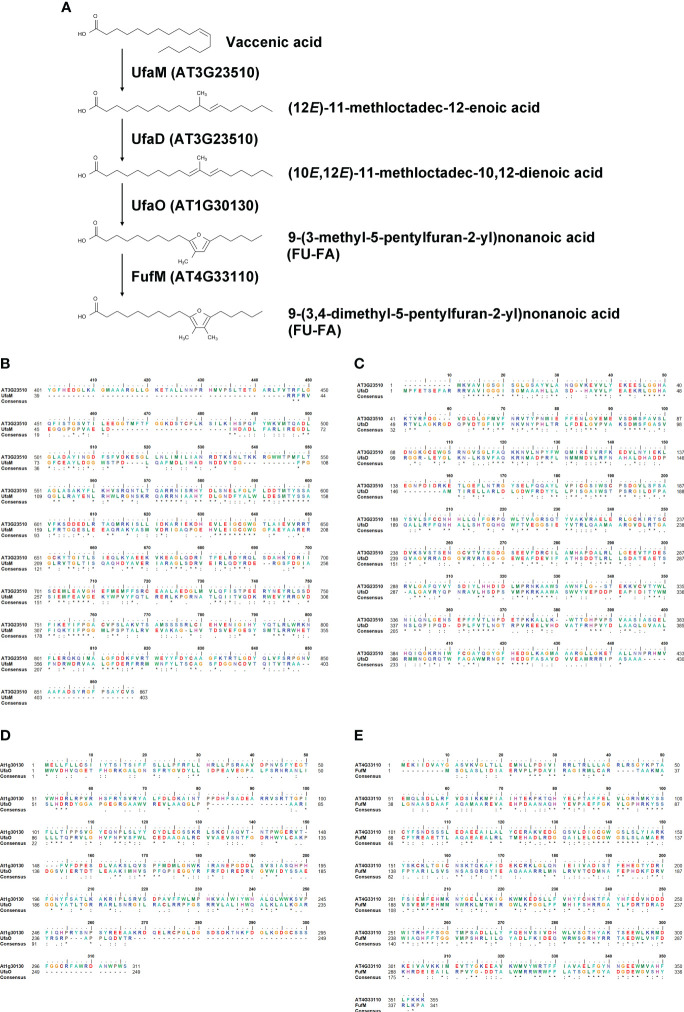
Homology search of LD-associated proteins AT3G23510, AT1G30130, and AT4G33110. **(A)** A furan-containing fatty acid biosynthesis pathway. **(B–E)** Amino acid sequence alignments between *A. thaliana* and *C. sphaeroides*: AT3G23510 and UfaM **(B)**; AT3G23510 and UfaD **(C)**; AT1G30130 and UfaO **(D)**; and AT4G33110 and FufM **(E)**.

There are currently no reports on the functions of the two proteins encoded by AT3G23510 and AT1G30130. To characterize their functions, we performed homology searches using the Basic Local Alignment Search Tool (BLAST) at the National Center for Biotechnology Information (NCBI). Our similarity searches revealed that amino acid sequences of the two proteins were highly similar to those of enzymes involved in furan-containing fatty acid (FU-FA) biosynthesis (**Figure 5A**) in the photosynthetic bacterium *Cereibacter sphaeroides* (Lemke et al., 2020). The amino acid sequence identity of AT3G23510 and UfaM was 34.26%, that of AT3G23510 and UfaD was 38.50%, and that of AT1G30130 and UfaO was 26.42% using UniProt (The Uniprot, 2017) (**Figures 5B–D**). Additionally, our similarity searches revealed that the amino acid sequence of FufM was highly similar to that of AT4G33110, with 37.87% amino acid sequence identity (The Uniprot, 2017) (**Figure 5E**). AT4G33110 (named LD METHYLTRANSFERASE1, LIME1) is classified as an S-adenosyl-L-methionine-dependent methyltransferase superfamily protein and localizes to LDs (Kretzschmar et al., 2020). We confirmed that LIME1-GFP localized to LDs (**Supplementary Figure S5**). These results suggest that the enzymes for FU-FA biosynthesis exist in *A. thaliana* and localize to LDs. We named AT3G23510, AT1G30130, and AT4G33110 as AtUFAMD1, AtUFAO1, and AtFUFM1/LIME1, respectively.

## Discussion

In this study, we performed LD proteomics to identify leaf LD proteins. Among 3,206 candidate leaf LD proteins, we identified MYOB14, AT3G23510, and AT1G30130, which localize to LDs. Since LD localization of MYOB14, AT3G23510, and AT1G30130 has not been reported previously, our proteome data may include many unidentified LD-associated proteins. Our LD proteomics method and the resulting data are therefore useful for investigating leaf LD proteins.

**Figure 6:**
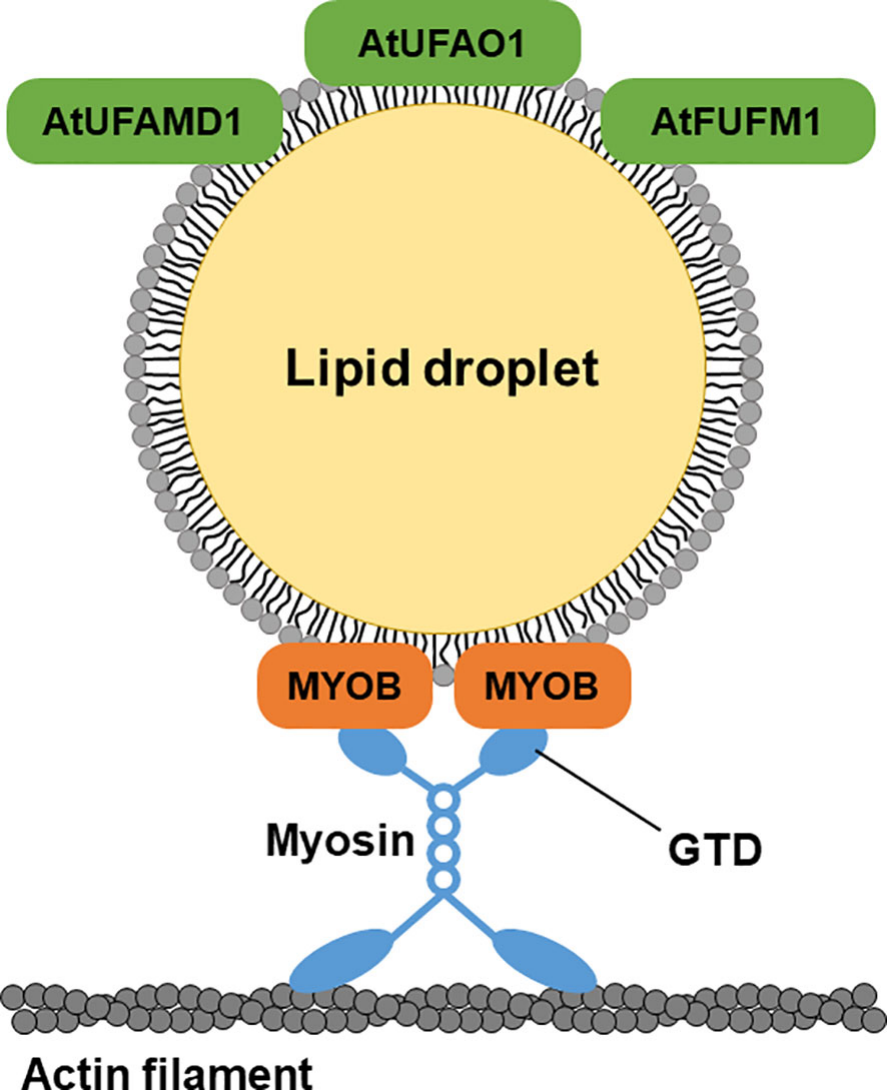
A hypothetical model of LD-localized proteins. LD-localized MYOBs for LD movement and LD-localized enzymes for furan-containing fatty acid biosynthesis.

LD movement is mediated by myosin in animals and plants (Pfisterer et al., 2017; Veerabagu et al., 2020; Han et al., 2023). MYOBs have a plant-specific DUF593 (Holding et al., 2007) and are involved in the movement of organelles in plant cells (Peremyslov et al., 2013; Peremyslov et al., 2015; Perico et al., 2021). However, the LD localization of MYOBs is unknown. MYOB1 and MYOB2 were previously reported to localize to motile vesicles (Peremyslov et al., 2013), and MYOB14 was reported to localize to uncharacterized compartments (Kurth et al., 2017). Here, we determined that MYOB1, MYOB2, MYOB3, MYOB5, and MYOB14 localize to LDs in *A. thaliana*, indicating that these proteins may be involved in LD movement. Our microscopy observations revealed that LDs move along actin filaments, suggesting that this movement is mediated by myosin and MYOBs (**Figure 6**). In addition, we observed that LDs move together with the ER. Organelle dynamics of the ER are closely related to those of actin filaments (Ueda et al., 2010). Our data suggest that LD movement along actin filaments is mediated by myosin XI and LD-localized MYOBs.

*A. thaliana* has 16 MYOBs (Peremyslov et al., 2013; Perico et al., 2021), and we determined that 5 of these localize to LDs, suggesting that there might be various LD-localized MYOBs in plant cells. LD localization of MYOBs may play important roles in plant physiology. MYOBs are characterized into three groups (Peremyslov et al., 2013): MYOB1, MYOB2, MYOB3, and MYOB5 are in group I, and MYOB14 is in group III. Myosin XIK interacts with MYOB1, MYOB2, MYOB3, MYOB5, and MYOB14 in *A. thaliana* (Peremyslov et al., 2013; Kurth et al., 2017). We detected myosin XIK in our Co-IP assays using MYOB2-GFP (**Supplementary Table S2**) and MYOB14-GFP (**Supplementary Table S3**) as well as in our LD proteomics data (**Supplementary Table S1**). Thus, LD-localized MYOBs may function with myosin XIK in LD movement.

UfaM, UfaD, UfaO, and FufM are enzymes involved in FU-FA biosynthesis in the photosynthetic bacterium *C. sphaeroides* (Lemke et al., 2014;  Lemke et al., 2020; Yu and Shanklin, 2020). *A. thaliana* contains three proteins whose amino acid sequences are highly similar to those of the bacterial enzymes for FU-FA biosynthesis: AT3G23510 (AtUFAMD1), AT1G30130 (AtUFAO1), and AT4G33110 (AtFUFM1/LIME1). These proteins localize to LDs (**Figure 6**). FU-FAs have been detected in *Hevea brasiliensis* latex (Liengprayoon et al., 2011) and in soybean (*Glycine max*) and soy products (Müller et al., 2020); however, FU-FA biosynthesis in plants is largely unknown. Our findings indicate that FU-FA biosynthesis may occur in LDs. It will be interesting to investigate whether AtUFAMD1, AtUFAO1, and AtFUFM1/LIME1 directly function in FU-FA biosynthesis.

## Data availability statement

The original contributions presented in the study are included in the article/**Supplementary Material**. Further inquiries can be directed to the corresponding authors.

## Author contributions

YO: Conceptualization, Data curation, Formal analysis, Methodology, Project administration, Visualization, Writing – original draft. RS: Data curation, Formal analysis, Methodology, Visualization, Writing – original draft. EM-S: Data curation, Formal analysis, Methodology, Writing – original draft. KK: Data curation, Formal analysis, Methodology, Writing – original draft. HU: Conceptualization, Data curation, Formal analysis, Methodology, Project administration, Supervision, Writing – original draft. IH-N: Conceptualization, Data curation, Formal analysis, Project administration, Supervision, Writing – original draft. TS: Conceptualization, Data curation, Formal analysis, Funding acquisition, Methodology, Project administration, Resources, Visualization, Writing – original draft.

## Supplementary material

Supplementary Figure 1 Fluorescence images of *N. benthamiana* leaves transiently co-expressing α-DOX1-RFP and the GFP-fused candidate proteins.

Supplementary Figure 2 Fluorescence images of epidermal cells of true leaves of *N. benthamiana* transiently co-expressing α-DOX1-GFP (LDs, punctate structures) and mCherry-HDEL (ER, network structures).

Supplementary Figure 3 Co-immunoprecipitation using transgenic *A. thaliana* expressing MYOB2-GFP or MYOB14-GFP. **(A, B)** Fluorescence images of MYOB2-GFP **(A)** and MYOB14-GFP **(B)** in epidermal cells of true leaves of 2-week-old *A. thaliana* plants. Arrowheads indicate LDs. **(C, D)** Immunoblot analysis of the co-immunoprecipitates of MYOB2-GFP **(C)** and MYOB14-GFP **(D)** plants using anti-GFP antibody.

Supplementary Figure 4 Subcellular localization of myosin XIK and LDs. **(A)** Fluorescence images of *N. benthamiana* leaves transiently expressing GFP-XIKGTD along with α-DOX1-RFP. **(B)** Fluorescence images of myosin XIK-YFP and MDH (LDs) in the hypocotyls of XIK-YFP plants following the induction of LD formation with mevalonic acid.

Supplementary Figure 5 Subcellular localization of AT4G33110. Fluorescence images of *N. benthamiana* leaves transiently expressing AT4G33110-GFP along with α-DOX1-RFP.

Supplemental Movie S1 Fluorescence in Lifeact–Venus plants (for visualizing actin filaments) stained with BODIPY 493/503 for visualizing LDs.

Supplemental Movie S2 Fluorescence in *N*. *benthamiana* leaves transiently co-expressing α-DOX1-RFP for LD visualizing and mCherry-HDEL for ER visualizing.
