# Supplementary figures and images for: Lipid droplets in Arabidopsis thaliana leaves contain myosin-binding proteins and enzymes associated with furan-containing fatty acid biosynthesis
Source: Front Plant Sci. 2024 Mar 1;15:1331479. doi: 10.3389/fpls.2024.1331479 (PMC10940516; doi:10.3389/fpls.2024.1331479)

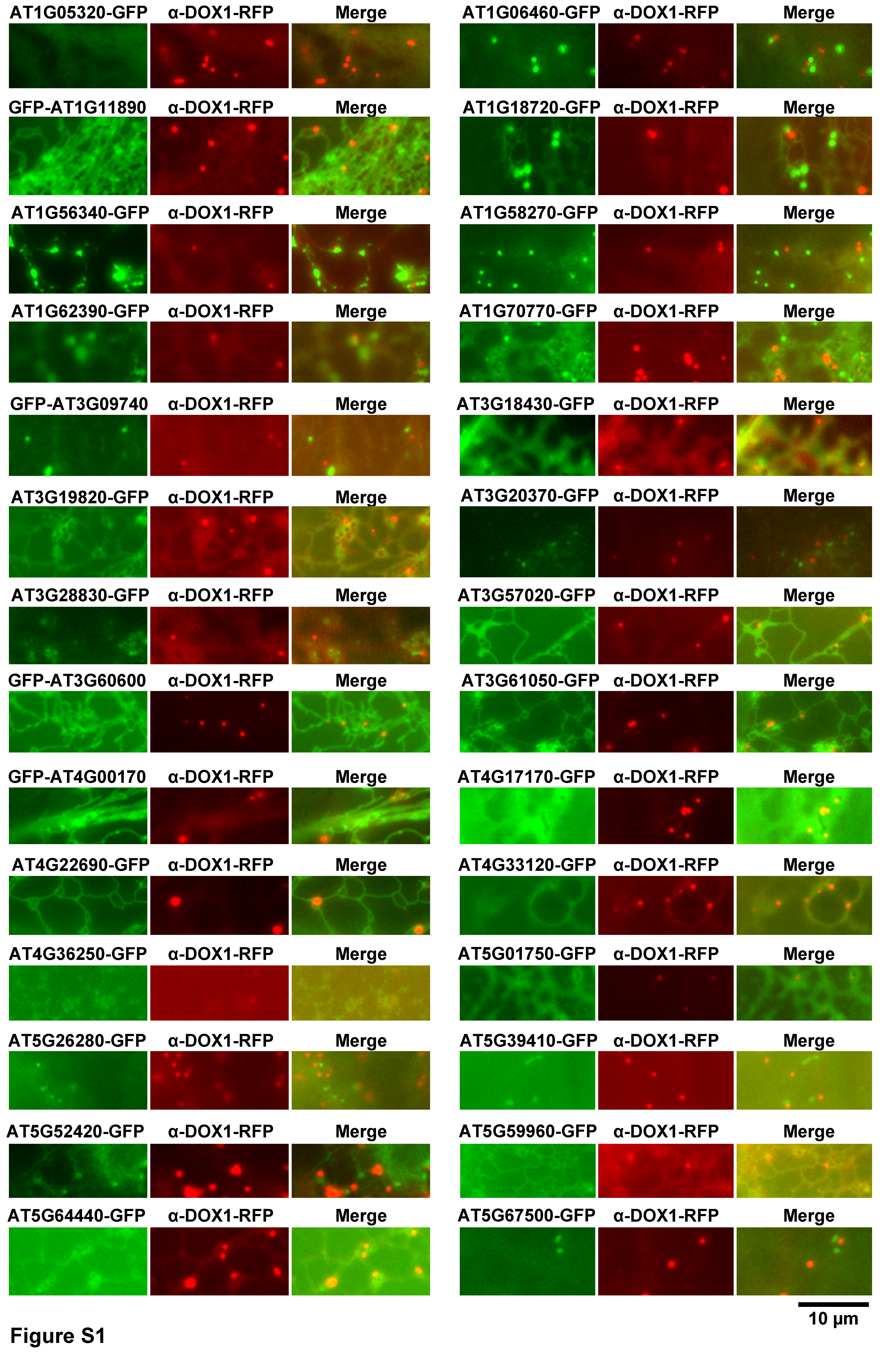

Supplement: Supplementary Figure 1 — Fluorescence images of N. benthamiana leaves transiently co-expressing α-DOX1-RFP and the GFP-fused candidate proteins. [file Image_1.tif]

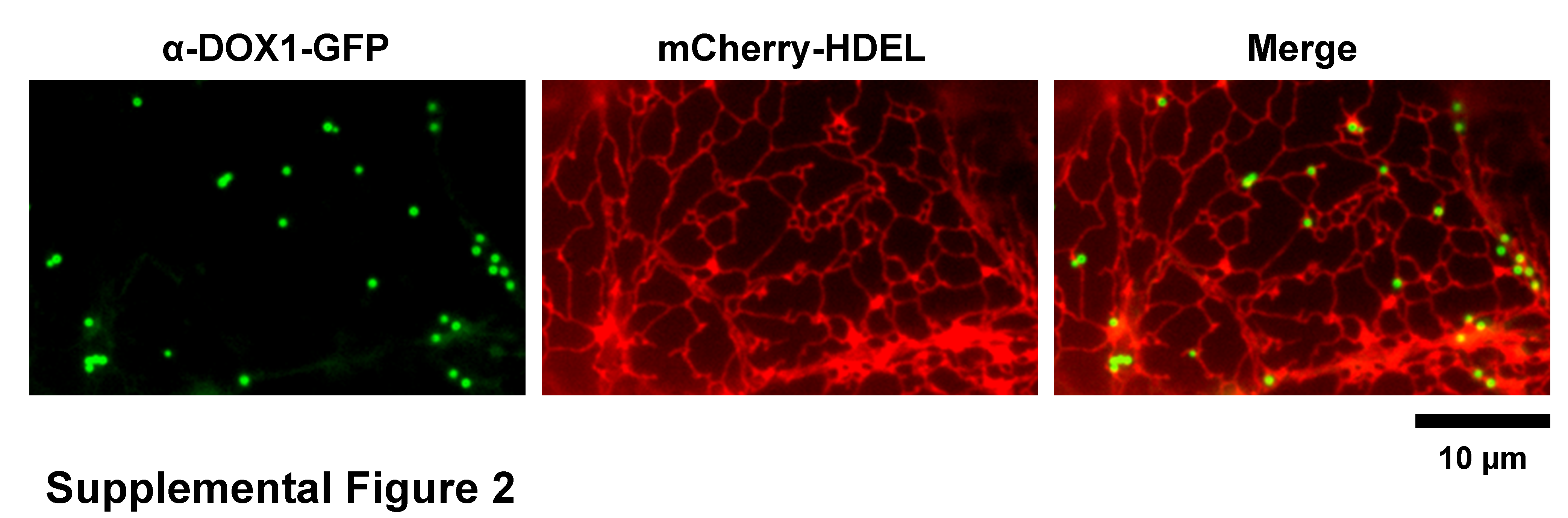

Supplement: Supplementary Figure 2 — Fluorescence images of epidermal cells of true leaves of N. benthamiana transiently co-expressing α-DOX1-GFP (LDs, punctate structures) and mCherry-HDEL (ER, network structures). [file Image_2.tif]

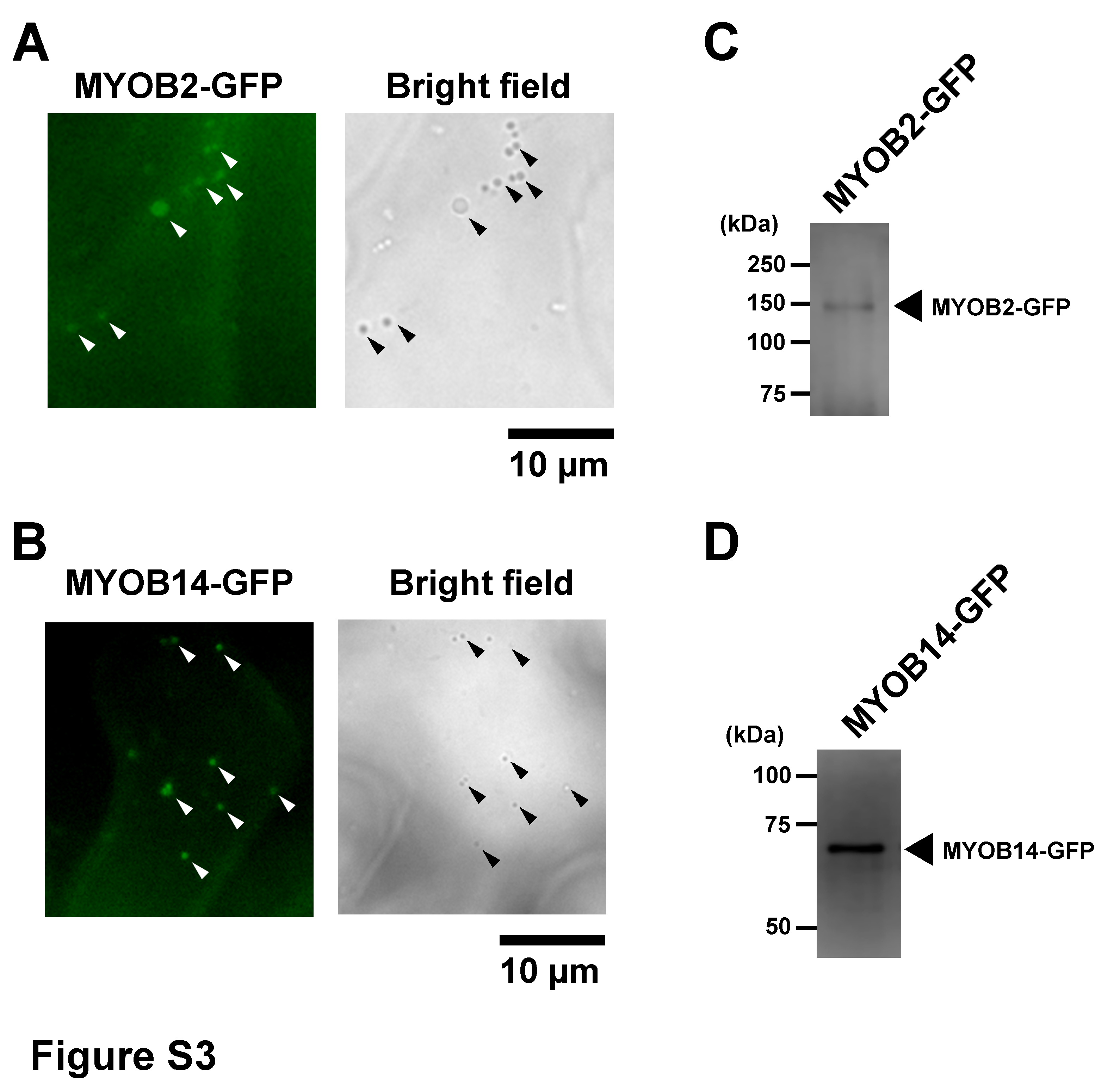

Supplement: Supplementary Figure 3 — Co-immunoprecipitation using transgenic A. thaliana expressing MYOB2-GFP or MYOB14-GFP. (A, B) Fluorescence images of MYOB2-GFP (A) and MYOB14-GFP (B) in epidermal cells of true leaves of 2-week-old A. thaliana plants. Arrowheads indicate LDs. (C, D) Immunoblot analysis of the co-immunoprecipitates of MYOB2-GFP (C) and MYOB14-GFP (D) plants using anti-GFP antibody. [file Image_3.tif]

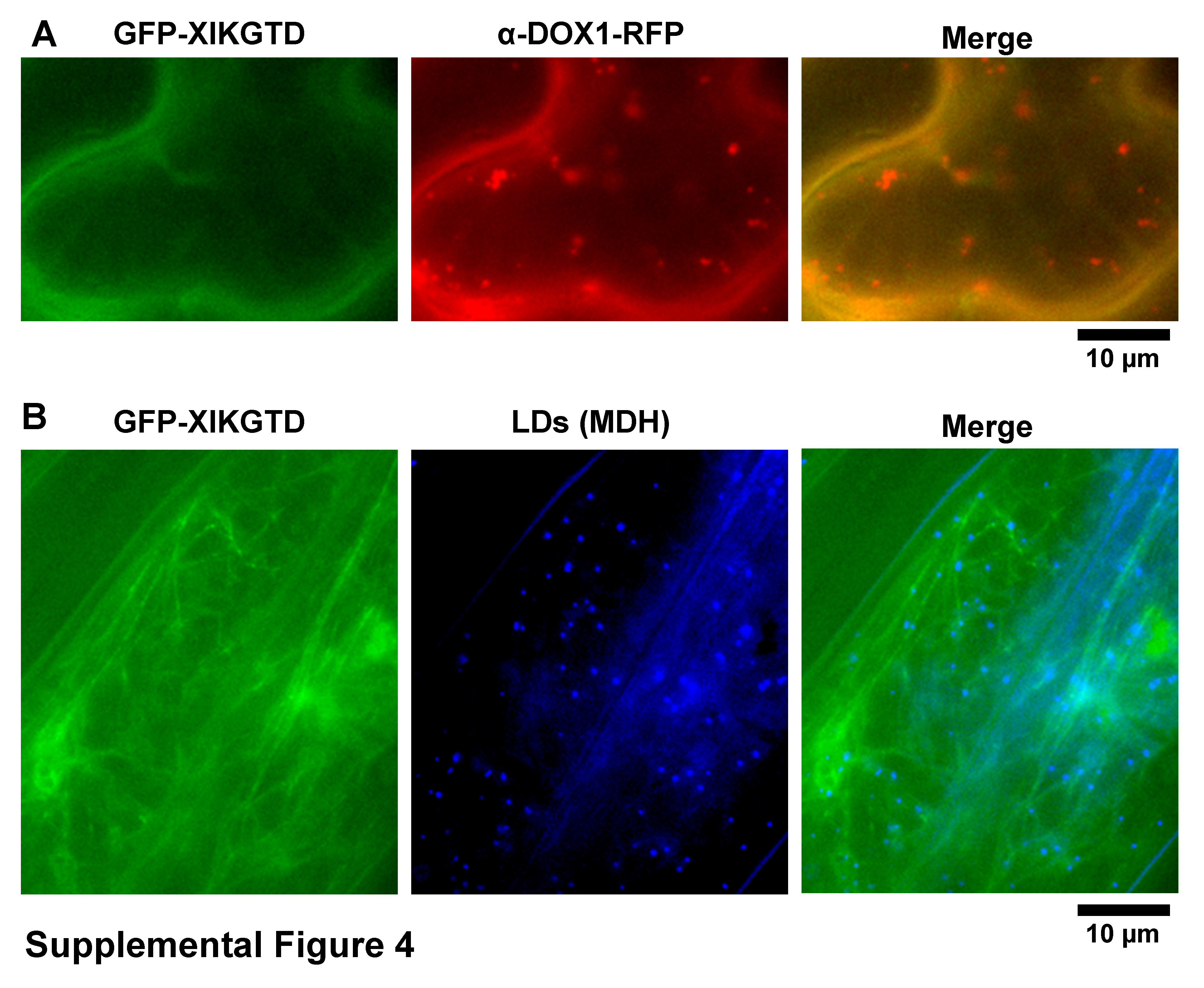

Supplement: Supplementary Figure 4 — Subcellular localization of myosin XIK and LDs. (A) Fluorescence images of N. benthamiana leaves transiently expressing GFP-XIKGTD along with α-DOX1-RFP. (B) Fluorescence images of myosin XIK-YFP and MDH (LDs) in the hypocotyls of XIK-YFP plants following the induction of LD formation with mevalonic acid. [file Image_4.tif]

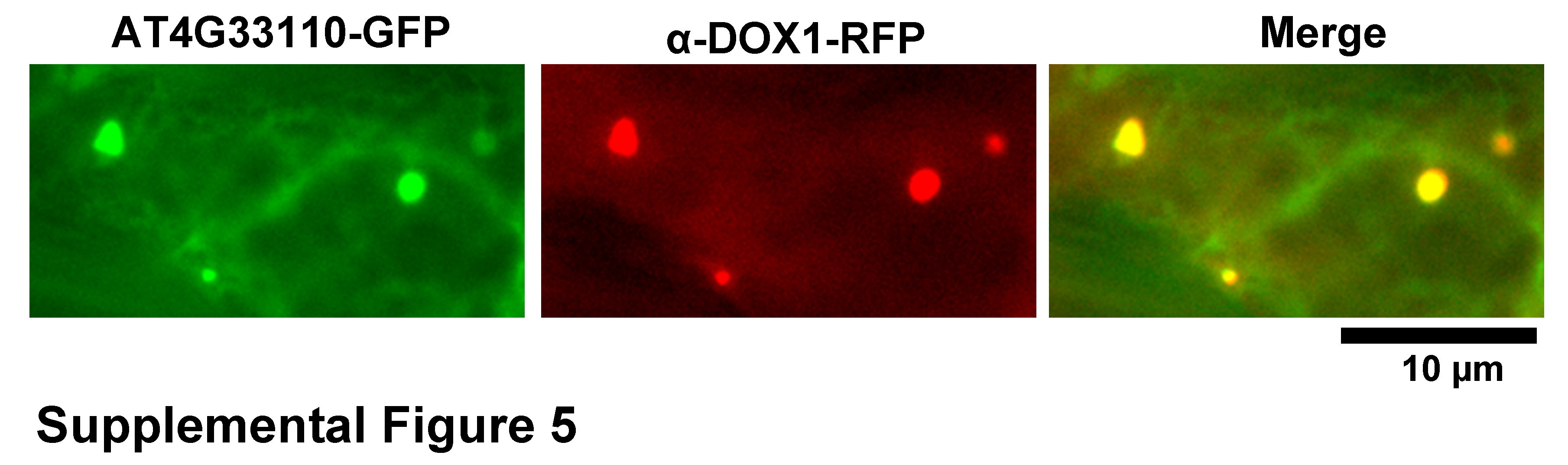

Supplement: Supplementary Figure 5 — Subcellular localization of AT4G33110. Fluorescence images of N. benthamiana leaves transiently expressing AT4G33110-GFP along with α-DOX1-RFP. [file Image_5.tif]
